# Prediction of trabecular meshwork-targeted micro-invasive glaucoma surgery outcomes using anterior segment OCT angiography

**DOI:** 10.1038/s41598-021-97290-8

**Published:** 2021-09-08

**Authors:** Yoko Okamoto, Tadamichi Akagi, Takanori Kameda, Kenji Suda, Masahiro Miyake, Hanako Ohashi Ikeda, Shogo Numa, Shin Kadomoto, Akihito Uji, Akitaka Tsujikawa

**Affiliations:** 1 Department of Ophthalmology and Visual Sciences, Kyoto University Graduate School of Medicine, Kyoto, Japan; 2 Division of Ophthalmology and Visual Science, Niigata University Graduate School of Medical and Dental Sciences, 1-757, Asahimachi-dori, Chuo-ku, Niigata, 951-8510 Japan

**Keywords:** Eye diseases, Ocular hypertension

## Abstract

We performed a prospective, longitudinal study to investigate the association between the preoperative intrascleral vasculature assessed using anterior segment (AS)-optical coherence tomography angiography (OCTA) and surgical outcomes of trabecular meshwork-targeted micro- or minimally invasive glaucoma surgery (MIGS). We included 37 patients with primary open-angle glaucoma. Preoperative AS-OCTA images of the sclero-conjunctiva of the nasal corneal limbus were acquired in the superficial (conjunctival) and deep (intrascleral) layers. The vessel densities (VDs) of each layer were measured separately in the entire area, limbal side, and fornix area. Surgical success was determined by postoperative intraocular pressure (IOP) and IOP reduction. Twenty-three and 14 eyes were classified as having successful and unsuccessful outcomes, respectively. The deep VDs of the entire area and fornix area were significantly lower in the successful group (*P* = 0.031 and *P* = 0.009). The success rate was significantly higher for eyes with a lower deep VD than for eyes with a higher deep VD. A greater IOP reduction was significantly associated with lower deep VD in the fornix area (*P* = 0.022) and higher preoperative IOP (*P* < 0.001). These results indicate that intrascleral vasculature assessed using preoperative AS-OCTA was negatively correlated with surgical success and IOP reduction resulting from trabecular meshwork-targeted MIGS. AS-OCTA images might help predict MIGS outcomes.

## Introduction

Glaucoma is characterized by progressive degeneration of retinal ganglion cells. Elevated intraocular pressure (IOP) occurs as a result of impaired aqueous humor outflow (AHO)^1^. The only established method of treating glaucoma is IOP reduction^2^. In the trabecular AHO pathway, the aqueous humor is drained from the trabecular meshwork (TM) through the Schlemm canal (SC), collector channels, and scleral and episcleral venous plexuses or aqueous veins, and then into the episcleral veins^1^. Although the TM is identified as the primary resistor to AHO in human eyes, resistance to post-TM outflow is also considered to affect AHO^1,3,4^.

TM-targeted micro- or minimally invasive glaucoma surgeries (MIGSs), such as trabectome surgery^5,6^, microhook ab interno trabeculotomy^7^, suture trabeculotomy^8,9^, and the use of Kahook dual blade^10^, have recently become popular as IOP-lowering treatments. These surgeries enable safe and quick relief of AHO resistance by cleavage or removal of the TM and inner walls of the SC; however, the variability and inconsistency in their IOP-lowering effects are problematic. Post-TM outflow resistance is thought to be one of their causes^4,11,12^; therefore, a clinically useful predictor of MIGS outcomes is necessary.

Recently, we reported that anterior segment optical coherence tomography angiography (AS-OCTA) images of the deep layer, which mainly comprise the intrascleral vasculature, can at least partly represent the post-TM AHO pathway; furthermore, vessel density (VD) in the deep layer was significantly associated with IOP in eyes that were treated for glaucoma^13,14^. Considering that vasculature images assessed using AS-OCTA reflect the post-TM AHO pathway, and that outcomes after MIGS may be influenced by the post-TM AHO, we hypothesised that preoperative AS-OCTA images would be related to the surgical outcomes of TM-targeted MIGS. In the present study, we prospectively examined the association between preoperative AS-OCTA images and the postoperative results of TM-targeted MIGS.

## Results

Forty-three eyes of 43 consecutive patients were enrolled in the study. Six eyes were excluded from the analysis because of poor-quality AS-OCTA images. The demographic and clinical characteristics of the 37 patients who were included are shown in Table 1. Postoperative hyphema and transient IOP spike were observed in 10 and 9 eyes, respectively, but no other serious postoperative complications were observed. Three patients were classified as the unsuccessful group because additional glaucoma surgeries were required 6 to 8 months after MIGS. Eventually, 23 eyes were classified as the successful group, and 14 eyes were classified as the unsuccessful group based on criterion A (Table 2). There were no significant differences in age, sex, axial length, central corneal thickness, preoperative IOP, number of preoperative anti-glaucoma eye drops, type of surgical procedure, or combination with cataract surgery between groups (all *P* > 0.05). The number of anti-glaucoma eye drops 1 year after surgery was significantly lower in the successful group than in the unsuccessful group (1.6 ± 1.3 vs. 3.3 ± 0.9; *P* < 0.001). The IOP at 1 year after surgery was significantly lower in the successful group than in the unsuccessful group (14.3 ± 2.7 mmHg vs. 18.9 ± 3.9 mmHg; *P* < 0.001). The rate of change in the IOP at 1 year after surgery was significantly lower in the successful group than in the unsuccessful group (− 37.7 ± 14.2% vs. 3.2 ± 24.1%; *P* < 0.001). The incidences of postoperative hyphema and IOP spike were not significantly different between the two groups (both *P* > 0.05).

**Table 1:** Demographic and clinical characteristics of the patients (N = 37).

Age (years)	71.8 ± 9.9 (44–89)
Sex (female/male), no	13/24
Diagnosis (POAG/PPG), no	35/2
Preoperative IOP (mmHg)	23.4 ± 7.1 (15–42)
Axial length (mm)	24.69 ± 1.53 (21.96–29.77)
CCT (μm)	528.4 ± 39.9 (435–615)
VF mean deviation (dB)	− 9.13 ± 5.47 (− 22.58–0.49)
Preoperative glaucoma eye drops, no	3.8 ± 0.9 (2–5)

Data (except sex and diagnosis) are presented as the mean ± standard deviation with the minimum and maximum values in parentheses.

CCT = central corneal thickness; IOP = intraocular pressure; POAG = primary open-angle glaucoma; PPG = preperimetric glaucoma; VF = visual field.

**Table 2:** Comparison between successful and unsuccessful groups (N = 37).

	Successful group (N = 23)	Unsuccessful group (N = 14)	*P* value
Age at baseline (years)	71.3 ± 10.6	72.6 ± 9.0	0.69
Sex (female/male), no	8/15	5/9	1.00^a^
Axial length (mm)	24.61 ± 1.42	24.84 ± 1.76	0.66
CCT (μm)	526.7 ± 32.7	531.1 ± 50.75	0.75
VF mean deviation (dB)	− 8.35 ± 5.61	− 10.39 ± 5.18	0.28
Single/Combined with cataract surgery, no	10/13	4/10	0.49^a^
Angle of trabeculotomy incision range (degree)	150.0 [100.0, 270.0]*	120.0 [97.5, 157.5]*	0.36^b^
Surgical procedure (microhook/TOM/s-LOT)	6/6/11	6/5/3	0.34^a^
Preoperative anti-glaucoma eye drops, no	3.5 ± 1.0	4.1 ± 0.6	0.072
Preoperative IOP (mmHg)	24.3 ± 7.6	21.9 ± 6.2	0.33
Anti-glaucoma eye drops at 1 year, no	1.6 ± 1.3	3.3 ± 0.9	** < 0.001**
IOP at 1 year (mmHg)	14.3 ± 2.7	18.9 ± 3.9	** < 0.001**
IOP reduction at 1 year (%)	37.7 ± 14.2	3.2 ± 24.1	** < 0.001**
Postoperative hyphema (yes/no)	7/16	3/11	0.71
Postoperative IOP spike (yes/no)	4/19	5/9	0.25
**Superficial layer**			
VD (entire area) (%)	27.67 ± 7.31	28.57 ± 8.52	0.73
VD (limbal area) (%)	21.91 ± 8.89	24.49 ± 7.38	0.37
VD (fornix area) (%)	31.43 ± 8.67	30.61 ± 10.63	0.80
**Deep layer**			
VD (entire area) (%)	10.00 ± 4.32	14.80 ± 8.61	**0.031**
VD (limbal area) (%)	15.05 ± 7.02	18.42 ± 11.08	0.26
VD (fornix area) (%)	7.50 ± 3.50	12.75 ± 8.01	**0.009**

Statistically significant values (*P* < 0.05) are shown in bold.

Data are presented as the mean ± standard deviation unless otherwise indicated.

*Data are presented as the median [25-th percentile, 75-th percentile].

CCT = central corneal thickness; IOP = intraocular pressure; s-LOT = suture trabeculotomy; TOM = Trabectome; VD = vessel density; VF = visual field.

*P*-value: ^a^Calculated using Fisher's exact test; ^b^Calculated using the Mann–Whitney U test; The other values were calculated using an unpaired *t-*test.

Success was defined as a postoperative IOP ≤ 18 mmHg and ≥ 20% reduction from preoperative IOP 1 year after surgery (criterion A).

The superficial VDs in the entire area, limbal area, and fornix area were not significantly different between the two groups (all *P* > 0.05). The deep VDs in the entire and fornix areas were significantly lower in the successful group than in the unsuccessful group (entire area: 10.00 ± 4.32% vs. 14.80 ± 8.61%, *P* = 0.031; fornix area: 7.50 ± 3.50% vs. 12.75 ± 8.01%, *P* = 0.009), whereas the difference in the deep VD in the limbal area was not significant (15.85 ± 7.92% vs. 17.19 ± 10.49%; *P* = 0.66).

Regarding criterion B, 24 eyes were classified as the successful group and 13 eyes were classified as the unsuccessful group. Similar to the results of criterion A, the deep VDs in the entire and fornix areas were significantly lower in the successful group than in the unsuccessful group (entire area: 10.13 ± 4.27% vs. 14.94 ± 8.95%, *P* = 0.033; fornix area: 7.67 ± 3.52% vs. 12.84 ± 8.33%, *P* = 0.012), but not in the limbal area (15.08 ± 6.87% vs. 18.61 ± 11.51%; *P* = 0.25).

**Figure 1:**
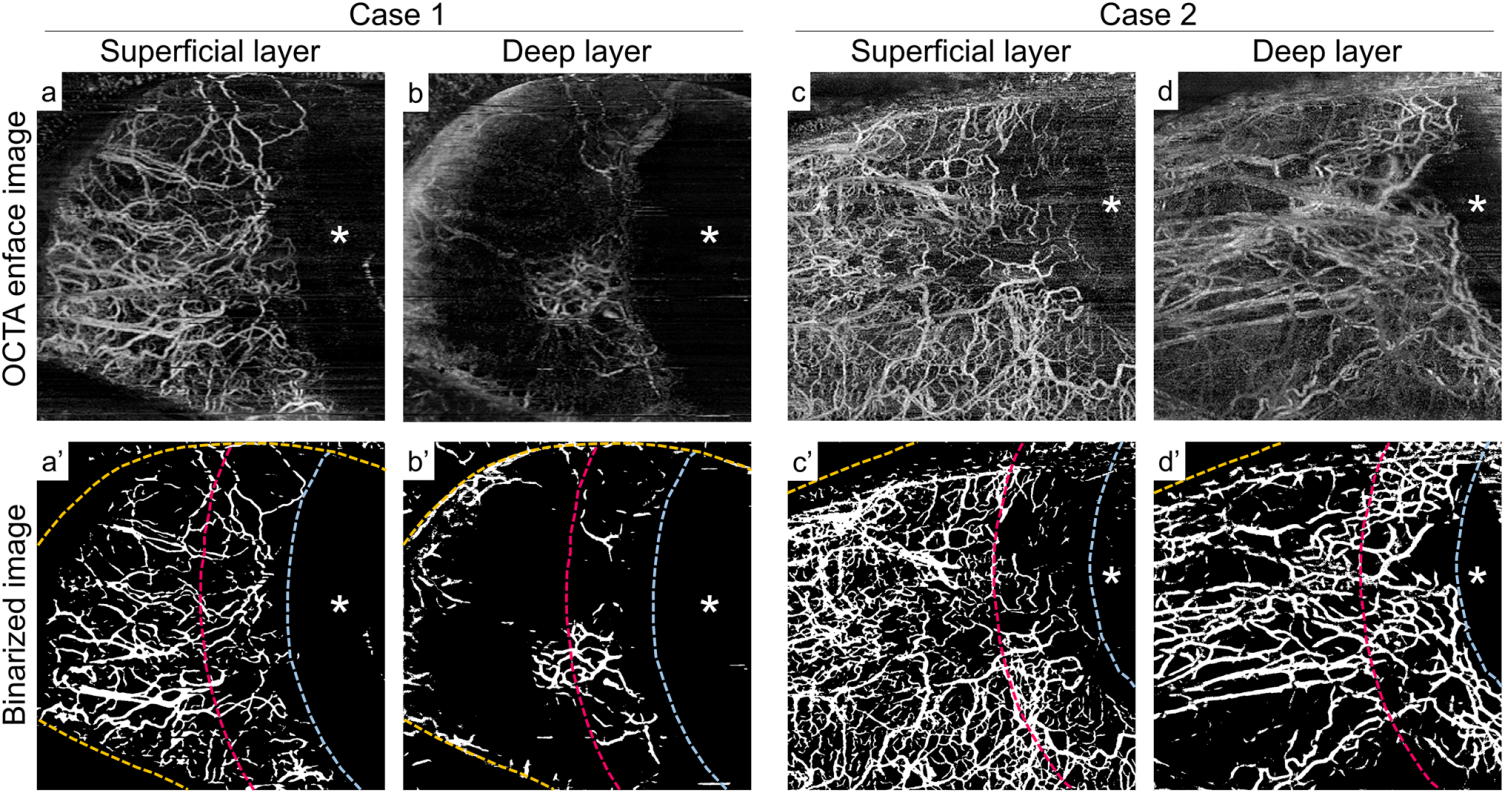
Representative anterior segment (AS) optical coherence tomography angiography (OCTA) images. (**a,b**) AS-OCTA images of a left eye in the successful group (case 1). (**c,d**) Mirror-reversed AS-OCTA images of a right eye in the unsuccessful group (case 2). (**a**′**–d**′) The binarized images of (**a**–**d**). The aqua dotted line is the boundary of the corneal limbus, and the red dotted line is the boundary between the limbal and fornix areas. The orange dotted lines are the eyelid margins. The flow signals in the deep layer are rich in the limbal area, although there are fewer in the fornix area (**b**,**b**′); however, flow signals in the deep layer are abundant in the fornix area as well as in the limbal area (**d**,**d**′). Asterisks indicate the location of the cornea.

AS-OCTA images of representative cases are shown in Fig. 1. For case 1 (the successful group according to criteria A and B), the superficial layer flow signals showed centrifugal patterns from the limbus (Fig. 1a), and the deep layer flow signals showing segmental patterns were low, especially in the fornix area (Fig. 1b). However, for case 2 (the unsuccessful group according to criteria A and B), a segmental pattern was disrupted, and flow signals were more abundant in the deep layer OCTA images, especially in the fornix area (Fig. 1d).

A logistic regression analysis was performed to examine the factors associated with surgical success based on criterion A (Table 3). A univariate analysis showed that the lower deep VDs in the entire area (*P* = 0.043) and fornix area (*P* = 0.021) were significantly associated with better surgical success. A multivariate analysis showed that lower deep VDs in the entire area (odds ratio [OR], 0.861; 95% confidence interval [CI], 0.749–0.990; *P* = 0.036) and fornix area (OR, 0.839; 95% CI, 0.721–0.976; *P* = 0.013) were significantly associated with better surgical success even after adjusting for preoperative IOP, the number of preoperative anti-glaucoma eye drops, and combination with cataract surgery. An analysis using criterion B showed results similar to those of the analysis using criterion A; the lower deep VDs in the entire area (*P* = 0.046) and fornix area (*P* = 0.024) were significantly associated with better surgical success according to the univariate analysis (OR, 0.869; 95% CI, 0.745–0.985; *P* = 0.042) and multivariate analysis (OR, 0.824; 95% CI, 0.703–0.966; *P* = 0.017).

**Table 3:** Logistic regression analyses investigating factors associated with surgical success (N = 37).

	Univariate analysis			Multivariate analysis*			Multivariate analysis*		
	OR	95% CI	*P* value	OR	95% CI	*P* value	OR	95% CI	*P* value
Superficial VD (entire area), per 1%	0.984	0.902, 1.075	0.73						
Superficial VD (limbal area), per 1%	0.962	0.887, 1.044	0.36						
Superficial VD (fornix area), per 1%	1.010	0.939, 1.086	0.79						
Deep VD (entire area), per 1%	0.885	0.786, 0.996	**0.043**				0.861	0.749, 0.990	**0.036**
Deep VD (limbal area), per 1%	0.956	0.884, 1.034	0.261						
Deep VD (fornix area), per 1%	0.849	0.740, 0.976	**0.021**	0.839	0.721, 0.976	**0.013**			
Combined cataract surgery (vs. single)	0.520	0.125, 2.157	0.37	0.524	0.066, 4.130	0.25	0.441	0.069, 2.809	0.39
Preoperative IOP, per 1 mmHg	1.054	0.950, 1.170	0.32	1.110	0.932, 1.322	0.43	1.069	0.917, 1.247	0.39
Preoperative anti-glaucoma eye drops, no. (per 1)	0.427	0.178, 1.022	0.056	0.571	0.206, 1.581	0.065	0.444	0.164, 1.201	0.11
Angle of trabeculotomy incision range, per 1°	1.005	0.997, 1.014	0.19						
Presence of postoperative hyphema	1.604	0.357, 8.718	0.55						
Presence of postoperative IOP spike	0.379	0.082, 1.760	0.22						
Age, per 1 year	0.986	0.919, 1.057	0.69						
Female sex (vs. male)	0.960	0.239, 3.853	0.95						
Axial length, per 1 mm	0.905	0.585, 1.400	0.65						
CCT, per 1 μm	0.997	0.980, 1.014	0.74						
VF mean deviation, per 1 dB	1.074	0.946, 1.219	0.27						

Statistically significant values (*P* < 0.05) are shown in bold.

CCT = central corneal thickness; CI = confidence interval; IOP = intraocular pressure; OR = odds ratio; VD = vessel density; VF = visual field.

*Multivariate analysis for combination with cataract surgery, preoperative IOP, and all variables with *P* < 0.1 in a univariable regression model. The deep VD in the fornix area and that in the entire area were separately analyzed because of the multicollinearity.

**Figure 2:**
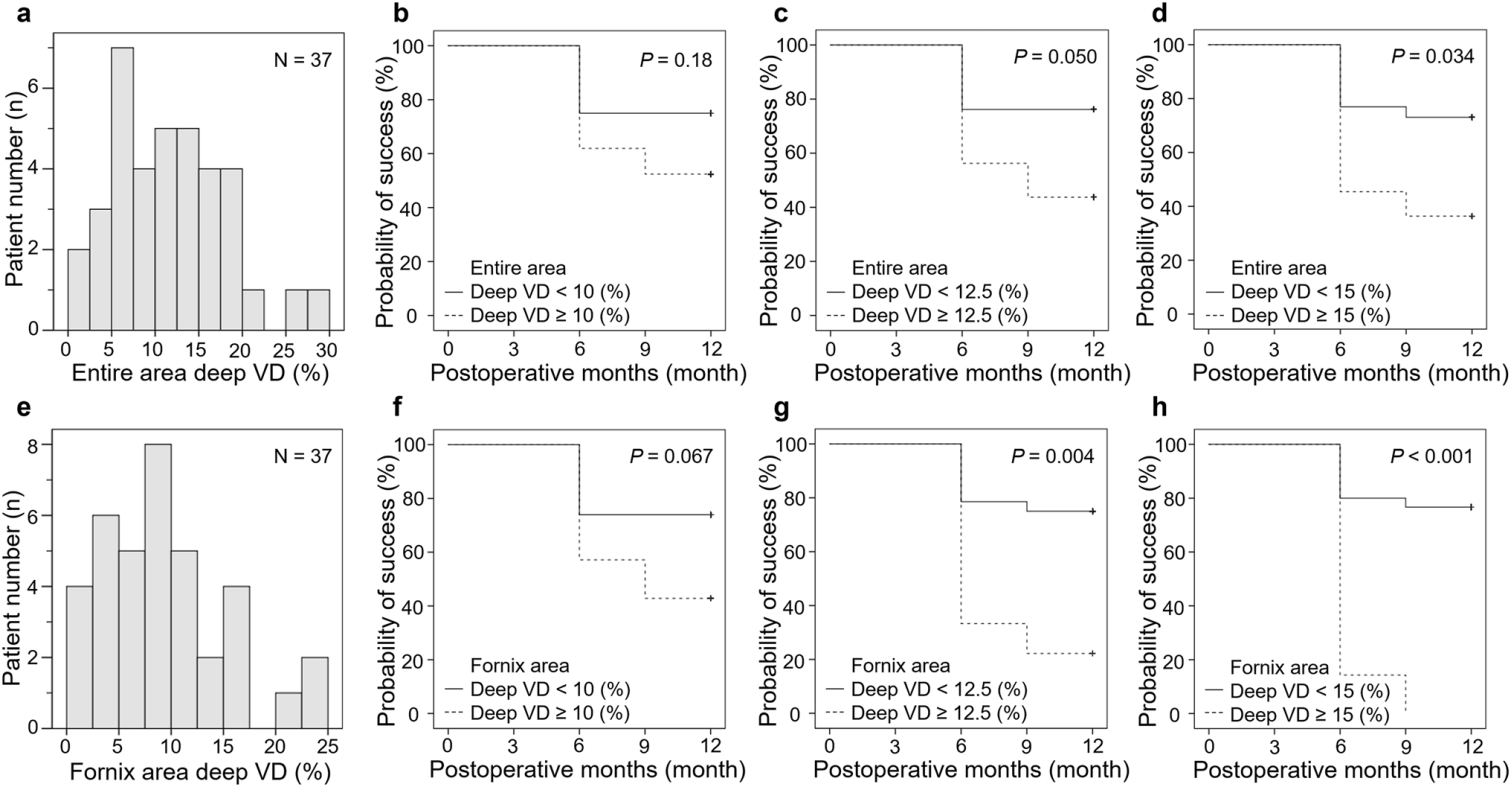
Kaplan–Meier cumulative survival curves of surgical success of two groups divided based on deep vessel density (VD) in the entire area and fornix area. (**a**) Histogram of the deep VDs in the entire area. (**b,c,d**) Kaplan–Meier cumulative survival curves of surgical success in the low VD group and high VD group classified according to the deep VD value (**b**: 10%; **c**: 12.5%; **d**: 15%) in the entire area. The low VD group had a higher success rate than the high VD group (**d**) (*P* = 0.034). (**e**) Histogram of the deep VDs in the fornix area. (**f,g,h**) Kaplan–Meier cumulative survival curves of surgical success in the low VD group and high VD group classified according to the deep VD value (**f**: 10%; **g**: 12.5%; **h**: 15%) in the fornix area. The low VD groups had higher success rates than the high VD group (**g**: *P* = 0.004; **h**: *P* < 0.001).

Kaplan–Meier cumulative survival analyses showed that the success rates for eyes with lower deep VDs were significantly higher than those for eyes with higher deep VDs in the entire area with reference to 15% (*P* = 0.034), and in the fornix area with reference to 12.5% (*P* = 0.004) and 15% (*P* < 0.001) (Fig. 2). However, the success rates were not significantly different between the eyes with lower and higher deep VDs in the limbal area with reference to 10%, 12.5%, and 15% (all *P* > 0.05; data not shown).

Factors associated with the percent changes in IOP were examined using multiple linear regression analyses (Table 4). The deep VD in the fornix area (B, 1.486; 95% CI, 0.227–2.745; β = 0.343; *P* = 0.022) and preoperative IOP (B, − 2.083; 95% CI, − 2.999 to − 1.168; β =  − 0.621; *P* < 0.001) were significantly associated with the percent changes in IOP.

**Table 4:** Factor associated with the percent changes in intraocular pressure (IOP) (N = 34).

	Univariate analysis			Multivariate analysis*		
	B (95% CI)	β	*P*	B (95% CI)	β	*P*
Superficial VD (entire area), per 1%	0.057 (− 1.168, 1.281)	0.017	0.93			
Superficial VD (limbal area), per 1%	0.166 (− 0.904, 1.236)	0.056	0.75			
Superficial VD (fornix area), per 1%	− 0.145 (− 1.127, 0.838)	− 0.053	0.77			
Deep VD (entire area), per 1%	0.885 (− 0.500, 2.271)	0.224	0.20			
Deep VD (limbal area), per 1%	0.181 (− 0.859, 1.221)	0.063	0.73			
Deep VD (fornix area), per 1%	1.445 (− 0.024, 2.914)	0.334	0.053	1.486 (0.227, 2.745)	0.343	**0.022**
Combined with cataract surgery (vs. single)	− 7.172 (− 15.730, 1.386)	− 0.289	0.098	− 3.557 (− 9.958, 2.844)	− 0.143	0.26
Preoperative IOP, per 1 mmHg	− 2.157 (− 3.081, − 1.232)	− 0.643	** < 0.001**	− 2.083 (− 2.999, − 1.168)	− 0.621	** < 0.001**
Preoperative anti-glaucoma eye drops, no	6.053 (− 2.759, 14.865)	0.240	0.17	2.372 (− 3.891, 8.636)	0.094	0.44
Angle of trabeculotomy incision range, per 1°	− 0.102 (− 0.194, − 0.010)	− 0.369	**0.032**	− 0.019 (− 0.106, 0.068)	− 0.067	0.66
Presence of postoperative hyphema	− 1.768 (− 11.429, 7.894)	− 0.066	0.71			
Presence of postoperative IOP spike	− 3.147 (− 13.650, 7.357)	− 0.107	0.55			
Age-baseline, per 1 year	0.490 (− 0.426, 1.406)	0.189	0.28			
Female sex (vs. male)	− 0.478 (− 0.416, 8.459)	− 0.019	0.91			
Axial length, per 1 mm	4.532 (− 0.809, 9.873)	0.292	0.094	3.374 (− 0.811, 7.559)	0.218	0.11
CCT, per 1 μm	0.110 (− 0.100, 0.320)	0.185	0.29			
VF mean deviation, per 1 dB	− 0.852 (− 2.514, 0.811)	− 0.181	0.30			

Statistically significant values (*P* < 0.05) are shown in bold.

Percent changes in IOP = (postoperative IOP 1 year after the surgery-preoperative IOP)/preoperative IOP × 100.

B = unstandardized regression coefficient; β = standardized regression coefficient.

CCT = central corneal thickness; CI = confidence interval; VD = vessel density; VF = visual field.

*Multivariate analysis for combination with cataract surgery, preoperative anti-glaucoma eye drops, and all variables with *P* < 0.1 in a univariable regression model.

## Discussion

This study showed that lower preoperative deep VD assessed using AS-OCTA was significantly associated with better surgical success and a greater IOP-lowering effect at 1 year after TM-targeted MIGS, especially in the fornix area. This association was significant even after adjustments for potential confounding factors, such as preoperative IOP and combination with cataract surgery. However, superficial VDs were not significantly associated with surgical success or IOP-lowering effects. The AS-OCTA deep flow signals of the vasculature, probably reflecting the function after TM pathway AHO, were thought to be related to the effects of MIGS.

Recently, visualization of the functional post-TM AHO pathway in the eyes of living human subjects has received attention^15–18^. IOP outcomes after MIGS were suggested to be associated with the visualized post-TM AHO; however, the aforementioned methods are relatively invasive, can be difficult to quantitatively evaluate the functional AHO, and should be performed under non-physiologic conditions in an operating room. AS-OCTA, as used in this study, is a relatively new technology that can noninvasively visualize iris neovascularization^19,20^, filtering bleb vascularization^21,22^, and the sclero-conjunctival vasculature^13,14,23,24^. Previous reports have shown that AS-OCTA could be used to visualize the post-TM AHO pathway, and that the OCTA VD in the deep layer was significantly associated with IOP in eyes treated for glaucoma^13,14^. Although AS-OCTA signals are derived from moving red blood cells and not from AHO^25^, AS-OCTA is advantageous because it is noninvasive and can save time. Additionally, it may be used under physiological conditions and for quantitative analyses.

The preoperative OCTA VD in the deep layer was significantly associated with the success rate and IOP-lowering rate (independent of preoperative IOP values) during the current study. These results suggest that lower OCTA VD in the deep layer can be used as a predictor of the success of MIGS. However, the mechanism of this association is unclear. MIGS procedures require a healthy downstream collector system and normal episcleral vein pressure to work effectively^26^. Increased episcleral venous pressure (EVP) may be one of the causes of MIGS failure. Our results suggest that low intrascleral OCTA signals, especially at the fornix area rather than at the limbal area, may indicate a healthy AHO pathway and normal EVP. Glaucoma caused by Sturge-Weber syndrome in patients with late-onset glaucoma is known to be related to increased EVP^27^. Zhao et al^28^ investigated the deep vasculature in the corneal limbus with AS-OCTA in Sturge-Weber syndrome patients and found that it was significantly associated with increased IOP, which was consistent with our results. Another possible explanation might be related to the pulsatile AHO. Pulsatile AHO has an important role in normal IOP maintenance and diminishes with glaucoma^29,30^. The episcleral or intrascleral vasculature with impaired pulsatile AHO might affect the AS-OCTA images, which detect moving red blood cells. Recently, we investigated the association between AS-OCTA images and the IOP-lowering effect of ripasudil instillation and found that lower deep VD was significantly associated with a greater IOP-lowering effect in normal eyes^23^. Ripasudil is a Rho-associated protein kinase inhibitor that lowers IOP by increasing AHO and reducing resistance in the TM. Because the IOP-lowering effect induced by ripasudil is thought to require good function of the post-TM AHO, this result is consistent with our results observed during the current study. Nonetheless, because the association between AHO function and AS-OCTA images has not been clarified, further detailed investigations are necessary.

The preoperative OCTA VDs in the superficial layer and deep limbal area were not significantly associated with the surgical outcome in the current study. The reason of the lack of association is unclear, but may be related to the number of vessels associated with AHO in the examined area. Because OCTA visualizes flowing red blood cells in both the veins and arteries, all vasculatures visualized by AS-OCTA are potentially not involved in AHO. Furthermore, some veins may not be involved in AHO. It is likely that the deep vasculature, particularly in the fornix area, is strongly associated with post-TM AHO.

During this study, patients underwent three different types of TM-targeted MIGS (trabectome surgery, microhook trabeculotomy, and suture trabeculotomy) depending on the surgeons’ preference. The success rate and IOP reduction rate observed during this study were not different among the different procedures. IOP reduction resulting from TM-targeted MIGS has been reported to occur approximately in the mid-teens regardless of the type of procedure^7,31,32^. Several reports have shown that the extent of incision or removal of the TM was not correlated with IOP reduction^32–35^, which was consistent with our results. However, a greater IOP reduction rate was significantly associated with higher preoperative IOP, which was also consistent with the results of previous studies^31,36,37^.

This study had some limitations. First, we could not completely exclude the possibility that IOP-lowering eye drops affected our results because several types of IOP-lowering eye drops were used before and after surgery. Nonetheless, during the current study, the number of anti-glaucoma eye drops administered was not significantly different for the successful and non-successful groups before surgery (Table 2). Furthermore, our previous study showed that the number of glaucoma medications was not significantly associated with deep VD in eyes treated for glaucoma^14^. Therefore, we believe that the effect of glaucoma medication had little impact on our results. Second, it is not easy to obtain high-quality AS-OCTA images. We excluded 6 of 43 examined eyes because of poor-quality AS-OCTA images. For four of the six excluded eyes, the background of the AS-OCTA images was whitish and showed unclear vasculature-like signals. Although we decided that these four images were of poor quality and had excessive noise and artifacts, it might be possible that these images had excessive AS-OCTA signals. However, it is probable that our results would not have been significantly affected if they had been included in the analysis because their surgical outcomes were unsuccessful. Future improvements in AS-OCTA equipment and software algorithms might lead to better usefulness of AS-OCTA. Third, the sample size was small and the follow-up period was short during this pilot study. Further investigations with larger sample sizes and longer follow-up periods are necessary to elucidate the potential use of AS-OCTA for long-term prediction of MIGS outcomes.

Currently, there is no useful method that can help predict MIGS outcomes. Our results elucidated the potential for AS-OCTA images to be used to predict MIGS outcomes, which can be beneficial for patients who require glaucoma surgery. Further large-scale investigations are needed before AS-OCTA can be accepted for clinical use.

## Material and methods

This study included patients who were enrolled in two ongoing prospective studies at Kyoto University Hospital: the Kyoto University Glaucoma Progression Study (registered with the University Hospital Medical Information Network [UMIN] Clinical Trial Registry of Japan [UMIN000019854]) and the Clarification of Eye Diseases using OCTA (UMIN000028853). Both study protocols and data accumulation were approved by the Institutional Review Board and Ethics Committee of the Kyoto University Graduate School of Medicine and conformed to the tenets of the Declaration of Helsinki. After the study design and risks and benefits of participation were thoroughly explained, written informed consent was obtained from each study participant.

### Participants

The candidates for this study were patients with primary open-angle glaucoma, preperimetric glaucoma, or ocular hypertension who underwent TM-targeted MIGS at Kyoto University Hospital between 1 October 2017 and 31 October 2019. To be included, the candidates had to have an open angle on gonioscopy, best-corrected visual acuity of 20/40 or better at baseline, preoperative IOP of 15 mmHg or greater, no history of intraocular surgery other than cataract surgery, and no other ocular disease (excluding cataract). When both eyes met the inclusion criteria, the eye that was treated first was included in the analysis. The exclusion criteria were as follows: follow-up period less than 12 months after surgery and poor-quality AS-OCTA images.

The patients had undergone a preoperative comprehensive ophthalmic examination including slit-lamp and gonioscopic examinations, measurement of best-corrected visual acuity (using a 5-m Landolt chart), axial length (IOLMaster 500; Carl Zeiss Meditec, Dublin, California, USA), central corneal thickness (SP-3000; Tomey, Tokyo, Japan), Goldmann applanation tonometry, standard automated perimetry (Humphrey Visual Field Analyzer; Carl Zeiss Meditec) with the 24–2 Swedish interactive threshold algorithm standard program, circumpapillary retinal nerve fiber layer measurements using a Spectralis HRA + OCT scanner (Heidelberg Engineering, Heidelberg, Germany), and AS-OCTA examinations. Clinical information was collected during follow-up visits at 1, 3, 6, 9, and 12 months postoperatively. During each follow-up visit, IOP measurement results obtained using a Goldmann applanation tonometer, the number of postoperative anti-glaucoma eye drops, and any complications were recorded. Postoperative hyphema was defined as the formation of niveau and postoperative IOP spike as an IOP ≥ 30 mmHg within 1 month postoperatively.

### Surgical procedure

Patients underwent MIGS in the area including the nasal hemisphere of the eye with or without phacoemulsification and the intraocular lens implant. Any of the following MIGS procedures were performed: trabectome surgery, microhook trabeculotomy, or suture trabeculotomy. Standard sub-tenon anesthesia using 2% lidocaine was induced before the procedures. Trabectome surgery was performed through a 1.70-mm temporal corneal incision, and the TM and inner wall of the SC were ablated using a trabectome handpiece (NeoMedix, Tustin, CA, USA) nasally to form a 100° to 120° arc^5,6^. Microhook trabeculotomy was performed through a temporal corneal port, and the TM and inner wall of the SC were incised nasally using a straight hook (Tanito ab interno Trabeculotomy Micro-hook; Inami & Co., Ltd., Tokyo, Japan) to create a 100° to 120° arc^7^. During the suture trabeculotomy procedure, after the TM and inner wall of the SC at the nasal area were incised using a Tanito Micro-hook through a temporal corneal port, a 5-0 nylon was inserted in the SC and pulled out from the location where the nylon suture stopped, resulting in the creation of a 180° to 360° arc^8,9^. The choice of operative procedure was left to the discretion of the operator, and the angle of the incised or removed TM was recorded during the surgery. The SC in the nasal quadrant was opened to the anterior chamber, regardless of which method was chosen. Procedures were combined with cataract surgery for patients who had visually significant cataracts that required surgical treatment.

After the surgical procedure, three types of drops were prescribed: moxifloxacin (0.5% VEGAMOX; Novartis Pharma Co., Ltd., Tokyo, Japan), betamethasone sodium phosphate (0.1% Rinderon; Shionogi & Co., Ltd., Osaka, Japan), and pilocarpine hydrochloride (2% Sanpilo; Santen Pharmaceutical Co., Ltd., Tokyo, Japan); these were to be administered four times per day. For patients who underwent cataract surgery, bromfenac sodium hydrate (BRONUCK; Senju Pharmaceutical Co., Ltd., Osaka, Japan) was prescribed. Betamethasone sodium phosphate was changed to fluolometholone (0.1% Flumetholon; Santen Pharmaceutical Co., Ltd., Tokyo, Japan) a few days after surgery. Moxifloxacin was stopped 1 month after surgery. The dose of pilocarpine hydrochloride was gradually decreased until 3 months after surgery and then stopped. Fluolometholone and bromfenac sodium hydrate were stopped within 3 months after surgery. Anti-glaucoma eye drops were stopped after surgery and resumed when the postoperative IOP exceeded the target IOP of each case.

### Definition of surgical success

We defined the primary surgical success criterion as postoperative IOP ≤ 18 mmHg and ≥ 20% IOP reduction from preoperative IOP (criterion A). Another criterion was IOP ≤ 18 mmHg and ≥ 20% IOP reduction from preoperative IOP or ≥ 10% IOP reduction with a reduction of ≥ 2 in the number of anti-glaucoma eye drops (criterion B) to confirm the results of criterion A. The IOP values were evaluated at 3, 6, 9, and 12 months postoperatively. Treatment was determined to be a failure if the success criteria were not met during two consecutive visits after the third postoperative month. Surgical failure also included subsequent glaucoma surgery and loss of light perception. The percent change in IOP was evaluated at 12 months postoperatively.

### Anterior segment optical coherence tomography angiography examination

The AS-OCTA examinations were performed before surgery using a swept-source OCT (PLEX Elite 9000) system with a 10-diopter optical adaptor lens developed by Carl Zeiss Meditec as previously reported^13,14,19,21^. A 3- × 3-mm scan was used to acquire the AS-OCTA images, which comprised 300 A-scans per B-scan repeated four times at each of the 300 B-scan positions. The AS-OCTA images were acquired in the nasal and temporal sclero-conjunctivas around the limbus, and the nasal images were used for the analysis because the TM was incised/removed in the nasal quadrant during MIGS in this study for all cases. En face images were generated using a built-in software (version 1.6; Carl Zeiss Meditec). The superficial layer (from the conjunctival epithelium to a depth of 200 μm) mainly comprised the conjunctiva, and the deep layer (from a depth of 200 μm to a depth of 1000 μm) mainly comprised the sclera. Artifacts caused by superficial images were removed from the deep images using the projection-resolved algorithm in the built-in software.

### Measurement of anterior segment optical coherence tomography angiography parameters

The VD was measured using an en face image of the superficial and deep layers in the nasal region. Vessel binarization was performed using the Trainable Weka Segmentation plugin in Fiji (free downloadable software, https://fiji.sc) to suppress the influence of noise in the AS-OCTA images^38–40^. First, we converted the original images to an 8-bit grayscale image using Fiji^38^. A representative OCTA image was used to train the software to classify vessels from the background by drawing representative lines inside and outside the selected vessels. After confirming the adequacy of vessel segmentation in the entire area of the image, the classifier was applied to all other images. Based on the binarized image, VD was shown as a ratio of pixels representing vessels of the entire area. During a previous study, the superficial and deep VDs in the AS-OCTA images were successfully calculated using this binarization algorithm^23^.

**Figure 3:**
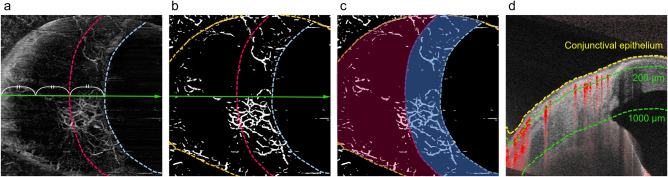
Measurement of vessel density using anterior segment (AS) optical coherence tomography angiography (OCTA) images. (**a**) A deep-layer AS-OCTA image of the nasal side around the corneal limbus. (**b**) A binarized image of (**a**). The aqua dotted line is the corneal marginal line, and the orange dotted lines are the eyelid margins. The red dotted line is a boundary between the limbal area (inner one-third region) and fornix area (outer two-thirds region). (**c**) The blue and red translucent areas are the limbal and fornix areas, respectively. The entire area is the sum of the limbal and fornix areas. (**d**) A cross-sectional AS-OCTA image overlying the B-scan image acquired at the green line in (**a**) and (**b**). The yellow dotted line is the conjunctival epithelium, and the green dotted lines are at depths of 200 µm and 1000 µm, respectively, from the conjunctival epithelium. The superficial layer (from the conjunctival epithelium to a depth of 200 µm) and deep layer (from a depth of 200 µm to 1000 µm from the conjunctival epithelium) mainly comprise the conjunctiva and sclera, respectively.

The VDs in the superficial and deep layers were calculated for the entire area, fornix area (inner one-third region), and limbal area (outer two-thirds region) in the nasal quadrant AS-OCTA image; the corneal region and eye lid area were excluded (Fig. 3). Anatomically, the AHO pathway in the sclera has a segmental pattern with a dense venous plexus around the limbus, which mainly comprises the intrascleral venous plexus and deep plexus. Some vessels extend towards the periphery and mainly comprise aqueous veins and the episcleral venous plexus^41^. Based on this distributional feature of the vasculature, we separately analyzed the limbal and fornix areas. The limbal area in the deep layer had a relatively rich vasculature in almost all cases. The corneal marginal line was determined as the line on the most corneal side where the flow signal existed (Fig. 3a,b, aqua dotted line). A point one-third from the corneal margin to the corner of the image (nose side) on a horizontal median line was defined, and a line with the same distance from the corneal margin was designated as the boundary line between the limbal and fornix areas (Fig. 3a,b, red dotted line). When the corneal region and eye lid margin were included in the image, they were carefully excluded from the analytical area (Fig. 3c).

### Statistical analyses

Categorical data are shown as numbers and percentages. Continuous data are reported as the mean ± standard deviation if the data had a normal distribution; if not, then the data are reported as the median and interquartile range. Normality of the data was assessed using the Shapiro–Wilk test and by visually inspecting histograms. Between-group comparisons of categorical variables were conducted using Fisher’s exact test. Between-group comparisons of continuous variables were performed using either an unpaired *t*-test for normally distributed data or a Mann–Whitney U test for nonparametric data. Factors for surgical success were analyzed using logistic regression models with forward selection, based on the likelihood ratio, or using the forced entry method, with significance determining the entry of variables. After dividing the eyes into two groups (low/high VD) using 10%, 12.5%, and 15% as boundary values based on the deep VD in the entire area and fornix area, comparisons of success rates between groups were evaluated using the Kaplan–Meier survival analysis and log-rank test. Factors affecting the rate of change in IOP were analyzed using linear regression models with a forced entry method, with significance for the entry of variables. All statistical analyses were performed using SPSS version 22.0 for Windows (IBM Japan, Tokyo, Japan). *P* < 0.05 was considered statistically significant.

## Data Availability

The raw data of the study are available at http://www.nature.com/srep.
